# The Meeting materials from the 4th Annual Meeting of the *International Society for the Prevention of Tobacco Induced Diseases*

**DOI:** 10.1186/1617-9625-3-1-42

**Published:** 2005-12-15

The meeting materials from the 4th Annual Meeting of the *International Society for the Prevention of Tobacco Induced Diseases* are available as a zip drive in additional file 1.

## Supplementary Material

Additional file 1 Click here for file

